# Gating by Memory: a Theory of Learning in the Cerebellum

**DOI:** 10.1007/s12311-021-01325-9

**Published:** 2021-11-10

**Authors:** Mike Gilbert

**Affiliations:** School of Psychology, University of Birmingham, Birmingham, UK

**Keywords:** Theory, Hypothesis, Learning, Cerebellum, Network, Circuit, Synapse

## Abstract

This paper presents a model of learning by the cerebellar circuit. In the traditional and dominant learning model, training teaches finely graded parallel fibre synaptic weights which modify transmission to Purkinje cells and to interneurons that inhibit Purkinje cells. Following training, input in a learned pattern drives a training-modified response. The function is that the naive response to input rates is displaced by a learned one, trained under external supervision. In the proposed model, there is no weight-controlled graduated balance of excitation and inhibition of Purkinje cells. Instead, the balance has two functional states—a switch—at synaptic, whole cell and microzone level. The paper is in two parts. The first is a detailed physiological argument for the synaptic learning function. The second uses the function in a computational simulation of pattern memory. Against expectation, this generates a predictable outcome from input chaos (real-world variables). Training always forces synaptic weights away from the middle and towards the limits of the range, causing them to polarise, so that transmission is either robust or blocked. All conditions teach the same outcome, such that all learned patterns receive the same, rather than a bespoke, effect on transmission. In this model, the function of learning is gating—that is, to select patterns that trigger output merely, and not to modify output. The outcome is memory-operated gate activation which operates a two-state balance of weight-controlled transmission. Group activity of parallel fibres also simultaneously contains a second code contained in collective rates, which varies independently of the pattern code. A two-state response to the pattern code allows faithful, and graduated, control of Purkinje cell firing by the rate code, at gated times.

**Supplementary Information:**

The online version contains supplementary material available at 10.1007/s12311-021-01325-9.

## Introduction

### Summary

This paper presents a theory of learning at the parallel fibre synapse which builds on previously published work [1, 2]. It is part of the wider proposal that the working of the circuit is not governed by a single overarching, unifying principle but is instead a suite of solutions to a number of design problems. Each paper covers a different aspect, written to stand alone.

The present paper, like the others, is in the form of a physiologically detailed hypothesis quantified by modelling. It argues that learning polarises synaptic weights, so that transmission is either robust or severely depressed, contrary to the traditional view that training teaches precision-graduated synaptic modification. The author has previously argued that parallel fibre activity contains two codes concurrently, a pattern code and a group rate code, contained in independent variables of the same parallel fibre activity [1]. The function of learning is gating [2]—pattern memory does not otherwise modify output. The gate has two states (functionally, a switch). This does not confine Purkinje cells to a binary response. On the contrary, it has the important (i.e., necessary) feature that, at gated times, interference by memory is *prevented*, so that transmission of the rate code is faithful and finely graduated.

The subject of this paper is synaptic modification by learning. This is complementary to the previously argued, functionally binary effect of pattern memory [2], but a different mechanism. Like the previous proposal, however, it operates at functionally unitary circuit level.

### Background

**Fig. 1:**
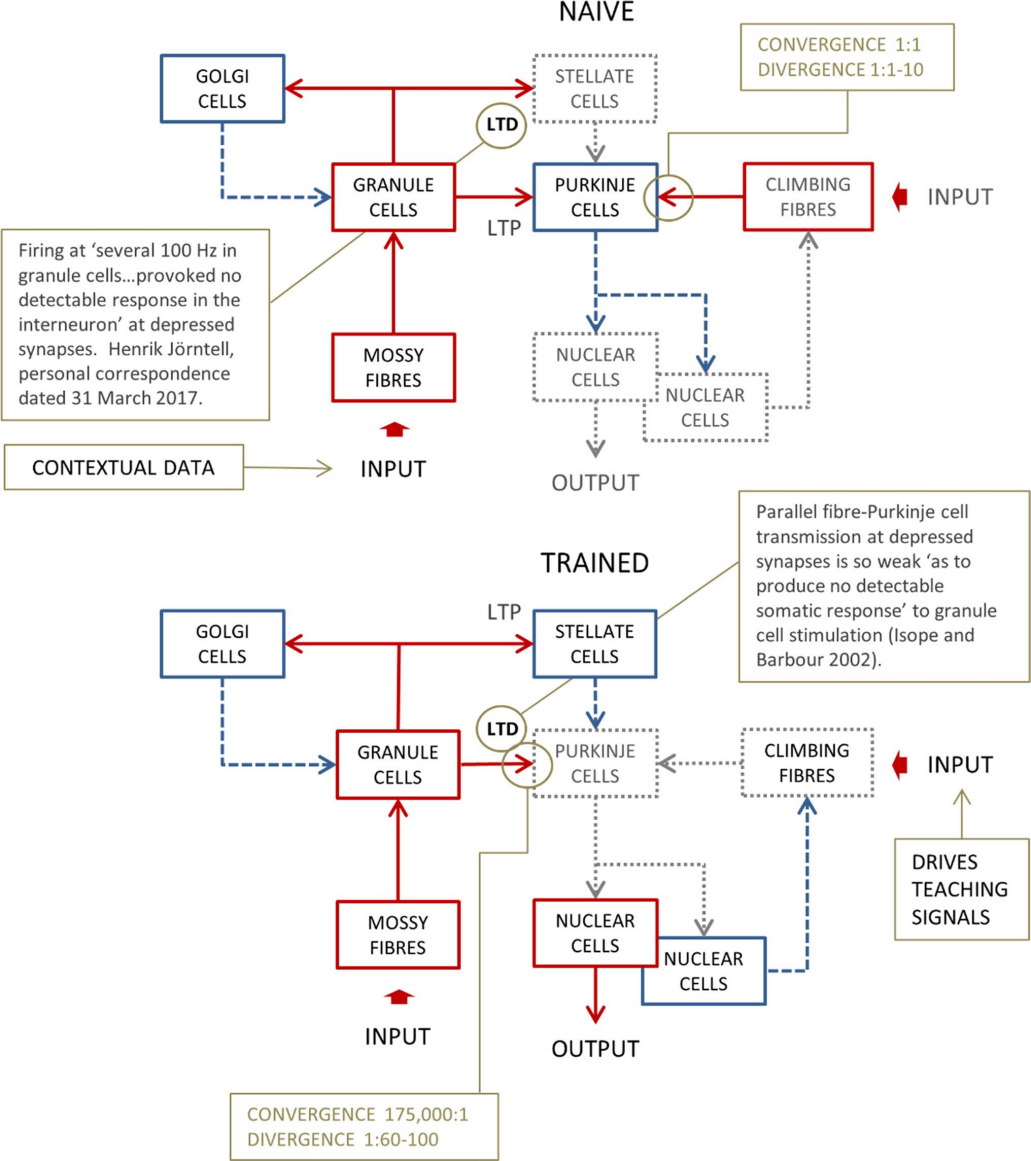
Schematic of circuit wiring. Each Purkinje cell receives intimate contact from a single climbing fibre, and a climbing fibre may contact 1–10 Purkinje cells. Microzones are defined by receiving input from functionally grouped climbing fibres which discharge together. Unlike mossy fibres, which are eclectically sourced (and more numerous), climbing fibres originate exclusively in the inferior olivary nucleus in the nearby brainstem. Pairing of parallel fibre synaptic activation with a convergent climbing fibre signal induces long-term depression of the parallel fibre-Purkinje cell synapse, i.e. enduring weakening of signal transmission. Synaptic transmission of granule cell signals to stellate cells is modified by training in the same conditions as the parallel fibre-Purkinje cell synapse but in the opposite direction. That is to say, transmission is strengthened by synaptic activation paired with a climbing fibre signal. Transmission at synapses not trained in this way is entirely absent, even tested with stimulation at hundreds of Hz

Parallel fibre synaptic activation that is repeatedly paired with a convergent climbing fibre signal induces long-term depression (LTD) of synaptic transmission at the parallel fibre-Purkinje cell synapse [3–5]. Enduring changes typically require repeated pairings—at least tens if not hundreds. The same conditions also induce modification of the parallel fibre-stellate cell synapse, but with the opposite sign, strengthening transmission [6–9] (long-term potentiation, LTP). (Fig. 1 is a schematic of the effect of learning on the pathway of signal transmission through the circuit.) The direction of learning at both synapse types is reversed when synaptic activation is not paired with a climbing fibre signal. Synaptic modification under climbing fibre instruction is involved in making new pathways which drive, for example, the conditioned blink reflex [originally: 10, 11].

It has been for years the central question of cerebellar theory, in what way and for what purpose do parallel fibre synaptic modifications change the Purkinje cell response? The attempt to understand the cerebellum has traditionally been dominated by a group of proposals collectively called the supervised learning model, which share some main features. The central premise is an assumption that the cerebellum implements a machine learning algorithm, so that the naive response to unknown input is displaced following training by a learned response to remembered patterns. Learning is stored as synaptic changes such that, following training, input to a Purkinje cell in a remembered pattern is passed through a corresponding set of precision-modified synaptic weights [12–15], strengthening transmission of some signals and weakening transmission of others. This has the result that the naive, unmodified response to input rates is displaced by a learned response which the pattern triggers. (A more in-depth commentary appears in the ‘Discussion’ section.) This proposal received early support from experimental confirmation of a role of climbing fibre signals in parallel fibre-Purkinje cell synaptic modification [16, 17] and from tracing of the conditioned eye blink reflex pathway through the cerebellum [10, 18]. An early and influential form of the model proposed that climbing fibre signals are triggered by behavioural errors [12], and this, too, seemed vindicated when it was reported that incorrect reaching movements by monkeys were associated with an increase in complex spikes, known to be a reliable indicator for climbing fibre discharge [19].

However, later evidence ultimately failed to provide corroboration of the expected role of LTD in motor learning [20–22], or that synaptic weights are functionally graduated [23], and more recently the notion that climbing fibre signals code errors has been called into question (see 24 for a review, which concludes they probably do not). For these and other reasons (see the ‘Discussion’ section), there are issues with the evidence for the old model, without naturally suggesting an alternative.

Evidence is not the only problem. It has never really been challenged, but is in fact not clear, whether a machine learning algorithm would be good at learning from real-world input data available to the cerebellum. In this context, ‘input data’ does not refer to individual signal metrics, or which particular cells are active, but other variables that affect the learning outcome, which override signal characteristics, so that they control the outcome.

For the system to reliably acquire a learned response to a remembered pattern, synaptic modification must tolerate overlap with other patterns. In unpaired trials, the direction of learning is reversed, so it must tolerate overlap with unpaired patterns too. Synapses are indefinitely plastic in both directions. The learning outcome is the result of a rolling history of relatively recent trials. At any given synapse, it depends ultimately on the ratio and sequence of synaptic activations which are paired with a climbing fibre signal (learning trials) and activations which are not (extinction trials). That depends in turn, at each synapse, on which patterns it is a member of, which of them occur in the relevant ‘period’ of recent history, how many times each, in what ratio with other patterns, and in what sequence.

A synapse may participate in a number of patterns (which may be all paired, all unpaired, or more likely a mixture). The number is controlled by a probability distribution—so the relative proportions that participate in 0 patterns, in 1, in 2, and so on is well predicted [2]—but the ratio and sequence are not, because real-world variables are not random and not predicted by fixed probabilities. As a result, each synapse is likely to receive a course of recent training which is for the most part independent of the training received at any other synapse. On the face of it, this should mean that the outcome at any particular synapse could feasibly be anything. That is not to say the outcome at synapses in a learned pattern is always or necessarily unrelated, but equally there is no reason why it always or necessarily would be, because there is no statistical safeguard against a noisy outcome.

### The Proposal

Yet a predictable outcome of training is a functional imperative. There cannot be uncertainty. The proposed cerebellar solution is that signals in a ‘remembered’ pattern are received at a representative random sample of learned synaptic weights, such that all learned patterns receive the same, rather than a bespoke, effect on transmission. In this model, learning implements and memory operates a pattern-activated gate in the form of a two-state balance of excitation and inhibition of Purkinje cells. There is not a graded effect of synaptic memory. Indeed, it is a necessary feature of this mechanism that it eliminates interference by weights with faithful transmission of rate coded signals. 

The mechanism is the physiological implementation of a function that describes a sigmoidal synaptic learning curve. The function is iterative—the size of change at each step depends on the output of the last, that is, on aggregate prior learning. This is because learning is calcium dependent and learned synaptic changes modify postsynaptic calcium amplitude.

Part of the proposal is that the shape of the learning curve is importantly functional, because it forces a well-predicted outcome regardless of input variables. To preserve the shape of the curve—to ring fence the relationship of learning and calcium—there is strict physiological control (by elimination) of other variables. This is the result, by design, of a number of features of cell anatomy and circuit architecture, giving a stripped-down character to the learning function.

The paper is accordingly divided into two parts. The first part is a physiological argument for the restriction of variables in the function and for the shape of the learning curve. The second part uses the learning function in a computational simulation to predict the outcome of learning across a population of synapses which are active in sparse sub-groups, in simulated real-world conditions.

### Methodology

The methodology is in some ways untypical for a network model. Network models often impose high-level ideas (and simplifying assumptions) top down onto what are in reality richly detailed biological systems. The present approach was in reverse, building with the evidence from the ground up and using modelling to test the ideas, so that the network level proposals are populated and supported by a detailed level of evidence.

## Physiological Model of Learning

### Design-Assisted Control of Variables in the Learning Function

This section argues for the physiological elimination of variables from the learning function, by cell and circuit design, thereby isolating an effect on calcium of learning itself.

#### The Parallel Fibre-Purkinje Cell Synapse

Stellate cell territories are flattened in the same plane as Purkinje cells, orthogonal to parallel fibres, so that they occupy the spaces between Purkinje cells [25]. Parallel fibres make contact in passing on stellate cells, as they do on Purkinje cells. The balance between excitatory and inhibitory input to a Purkinje cell is therefore affected by synaptic modification, which can be in both directions at both synapse types.

At the parallel fibre-Purkinje cell synapse, LTD involves removal by endocytosis of AMPA receptors from the postsynaptic membrane [26], and LTP their insertion [27], both calcium dependent. In mature Purkinje cells, most AMPA receptors contain the calcium-impermeable GluR2/GluR3 subunit. Calcium entry into the cell is through voltage-gated calcium channels [28–30] activated by AMPA-receptor-mediated depolarisation, so that learning acts on the conditions that teach it.

Parallel fibre-triggered calcium influx is locally confined [29]. Parallel fibres make contact on spines on thin distal branches of the Purkinje cell arbour, one per spine. A number of mechanisms combine to ensure that calcium elevation triggered by parallel fibre synaptic activation is confined to a spine-limited synapse-specific effect [29, 31]. Segregation of parallel fibre-evoked calcium transients is quite well understood [30].

Climbing fibre signals also trigger Purkinje cell dendritic calcium influx. Calcium transients peak at short latency with near simultaneous timing in all branches of the Purkinje cell dendritic arbour, including distal branches that bear spines [32]. While peak calcium transient amplitude varies with dendritic diameter and the density of calcium channels [33], these do not vary at the site of learning, in spine-bearing branchlets. So, timing and amplitude of transients do not vary between branches at the site of learning. Transients are separated by slow climbing fibre discharge rates [34], reflecting membrane properties [35], so do not overlap at typical rates, and do not sum anyway. ‘CS [complex spike]-evoked Ca^2+^ transients [are] a similar amplitude even when the dendritic Ca^2+^ level is already elevated’ in anaesthetised rats [32 p.10849]. Spines themselves share the same dimensions [31], so there is no variation of calcium concentration because of spine size. In theory, variation might result if climbing fibre input to a Purkinje cell on different occasions was to a variable number or permutation of synapses, or because it was from more than one climbing fibre, such that all synapses did not all receive the same signal. Potential variability in this way is absent because climbing fibre input to a Purkinje cell is from a single cell to all synapses simultaneously.

Climbing fibre signals themselves, which evoke dendritic transients, are in short bursts of a few spikes. These were originally reported to have an all-or-nothing signature [36] and then argued to code a variable lesson in the number of spikes they contain [37–39]. More recently it was counter-proposed that bursts contain a randomly variable and unreliably transmitted number of spikes which notify only whether a signal is present or not [2], arguing for a rehabilitation of the original view. This paper takes the view that instruction signals are functionally binary. As this is an important point but only briefly dealt with here, because the rationale has appeared previously [2], it is reproduced in Supplementary Materials (Supplementary Materials 1, ‘Climbing fibre instruction signals are not functionally variable’).

The significance of the foregoing remarks is that there is a systematic elimination of variables that could, and presumably would otherwise, affect postsynaptic calcium amplitude at the site of synaptic learning. This is achieved by a concerted effect of anatomy and electrophysiology—that is, by design. The parallel fibre transient is confined to a single spine, and each spine to contact from a single parallel fibre, and the climbing fibre transient peaks very fast with whole-arbour synchrony, and does not receive a variable effect in spine-bearing branchlets from dendritic transmission properties, signal frequency, or functional variation of the climbing fibre signal, which is received from a single cell.

#### The Parallel Fibre-Stellate Cell Synapse

There is less evidence for the same conclusion at the parallel fibre-stellate cell synapse than for the parallel fibre-Purkinje cell synapse, because there is less evidence generally, but what there is likewise suggests a pattern of adaptations that eliminate variables that affect calcium. Induction pathways in both directions are calcium dependent (for postsynaptic LTP, 6; for LTD, 7). ‘EPSCs at the parallel fibre to stellate cell synapse are predominantly mediated by AMPA receptors that lack GluR2 subunits’ and which are therefore permeable to calcium [40 p.559, citing 41]. Diffusion of dendritic calcium is heavily restricted [7], so that parallel fibre-triggered calcium is localised. Climbing fibres signal to stellate cells exclusively by glutamate spillover, which activates extrasynaptic receptors [42], so that there is a synchronised whole-arbour climbing fibre transient notwithstanding that dendrites do not all form part of the same connected tree. Plastic modulation of transmission is by competing induction pathways—blocking either reveals induction in the other direction [43].

### The Shape of the Learning Curve

Why is control of calcium important—what effect does calcium have on synaptic modification? There are thought to be two effects, on the direction and on the size of change, trial-by-trial.

#### Direction

The direction of change depends on the presence or not of a climbing fibre signal. To explain this, the idea of a calcium threshold [4] gained traction. In that proposal, the sum of calcium triggered by paired input is necessary to exceed the threshold for LTD, while a parallel fibre transient alone, below threshold, teaches LTP. This has been challenged. In vitro evidence suggests that high-frequency parallel fibre activation causes CaMKII phosphorylation which blocks LTD induction, preventing LTD induction by parallel fibre only activation [44]. LTD is blocked even at calcium amplitude that would otherwise induce it (of which parallel fibres are—otherwise—capable on their own). Climbing fibre instruction signals remove the block. ‘Climbing fibre co-activation prevents inhibitory autophosphorylation and restores LTD’ [44 p.13224].

This is not the only reason a climbing fibre signal is necessary for LTD. LTD is also dependent on corticotropin releasing factor, which is absent without climbing fibre signals [45], and on postsynaptic NMDA receptor activation at the climbing fibre-Purkinje cell synapse, which contributes to the climbing fibre-evoked calcium transient [46].

So, there is evidence that—and in the model—the direction depends on the presence, or not, of a climbing fibre signals, paired with synaptic activation, but the mechanism is not a calcium threshold. It is instead a block of phosphorylation and other agents.

#### Size

Assuming that, otherwise, induction in both directions scales with calcium amplitude according to some unknown function (which is feasibly but not necessarily linear), we can quantify synaptic change by making two predictions suggested by the evidence, and one which is unreported, as far as the author is aware. First, when a climbing fibre signal removes the block on LTD induction, LTP and LTD induction pathways are concurrently active and therefore in competition for calcium. In fact, this is reported for the parallel fibre-stellate cell synapse [43]. Second, the calcium supply is modulated by learning itself, because calcium entry into the postsynaptic cell is through or mediated by synaptic AMPA receptors. This is true for learning in both directions, in the presence of a climbing fibre signal or not. Third, the LTD induction pathway has higher affinity for calcium than the LTP induction pathway.

Together with the block of other variables, this is a physiological basis to propose that synaptic learning in both directions is described by an iterative sigmoid function, where per-step change accelerates after a slow start and then slows down towards a limit. Without a climbing fibre signal, the direction is always LTP, because parallel fibre-only activation causes CaMKII phosphorylation. When a climbing fibre signal is present, the direction is LTD because, with the block removed, the LTD induction pathway is more competitive, having a proposed higher affinity for calcium. The amount of change in each trial is a function of the last, because the aggregate learning outcome controls the calcium supply in the next.

This is not to overlook that induction is the outcome of complex molecular events. Rather, it is to propose that the outcome of those events is predicted by calcium amplitude and therefore by the effect on calcium of prior learning.

This paragraph is a hypothetical description of the mutual effect of calcium amplitude and learning across a course of training with all trials paired, at a previously severely depressed synapse. In early trials, calcium influx is weak because AMPA receptors are absent or sparse. Indeed, activation of AMPA receptors may be insufficient on its own to activate voltage-gated calcium channels, so that mGluRs may be necessary to assist calcium entry into the cell [29]. Therefore, LTP is in small early steps. As training proceeds, insertion of AMPA receptors into the postsynaptic membrane mediates stronger calcium influx, so that learning is in steps of increasing size. Ultimately, learning levels off where there is a diminishing return of more change. The limiting factor(s) may be a restriction on the density of AMPA receptors or the finite pool of calcium channels or an effect of calcium channel characteristics—T-type channels are only open in a limited, strongly polarised voltage range and then completely inactivated until the cell membrane repolarises [29], so that a fast-falling voltage would mean they are open for less time.

LTD may be described in similar terms, also hypothetical but based on the same evidence and predictions, starting this time with a robustly transmitting synapse. With the LTD block removed, change is the net outcome of competition for calcium. Early steps are small because calcium is abundant, so that LTP induction is barely affected by competition and keeps pace with LTD. The net outcome is still (because it is always, in paired trials) LTD [44], but initial progress is slow. In the middle range, the competition favours more competitive LTD because the calcium supply is progressively restricted and diminishing, so that net learning accelerates. Then, as the number of AMPA receptors becomes further depleted, in the later phase of training, the rate of receptor internalisation slows down again because calcium becomes severely depleted, so that the LTD pathway is also calcium-starved. This may be joined by other factors—the diminishing number of remaining AMPA receptors means there are fewer for the internalisation mechanism to act on, for example.

Both the description of LTP and of LTD describe only the artificial case where no trials are paired or they are all paired, respectively. But they provide the rationale to derive a model which can then be used to predict the outcome when training variables are generated trial by trial that simulate real-world conditions, which is to say, unpredictably and in no particular pattern.

## Computational Model of Learning

### The Core Model

To quantify these ideas, we might model trial-by-trial parallel fibre-Purkinje cell LTD induction as a sigmoid function of the presence, or not, of an instruction signal in that trial and the effect of learned change, as it is acquired, on the outcome of further training. The function is lean because instruction signals are not variable and because the effect of a parallel fibre signal on calcium in the postsynaptic spine is isolated by elimination of other variables. We might express this as:

$${L}_{n}={L}_{n-1}+k\left({T}_{n}-{L}_{n-1}\right)$$

where $L$ varies in the range $0<L<1$. ${L}_{n}$ is the outcome of trial $n$. A rising value of ${L}_{n}$ represents LTD. ${T}_{n}$ is either 1 (if there is a climbing fibre signal) or 0 (if there is not). $k$ is a constant, the learning coefficient, which adjusts Δ/trial (step size). $L$_lim→1_ is not where AMPA receptors are fully depleted, necessarily; it is where an effect of further training is exhausted. As noted, this (of course) belies the underlying complexity of molecular events, but because induction in both directions is calcium dependent, control of calcium predicts the outcome in the assumed absence of other (calcium independent) molecular variables.

So far, there is no early-stage inertia; nor is there an effect of parallel fibre signal frequency or of Purkinje cell dendritic membrane potential on calcium influx. These will all be added.

Adding early-stage inertia, ${L}_{n}$ is ${L}_{n-1}$ plus the product of

$$k\left({T}_{n}-{L}_{n-1}\right) and\left\{ \begin{array}{c} either\,{f}_{A}\left({L}_{n-1}\right), { T}_{n}=1\\ or\,{f}_{A}\left(1-{L}_{n-1}\right),{ T}_{n}=0\end{array}\right.$$

where ${f}_{A}\left(x\right)$ is a function which adjusts the strength of inertia, treated as a constant equal to 1. Signal frequency and membrane potential are added later.

### The Learning Coefficient: Repetition Is the Signal to Learn

**Fig. 2:**
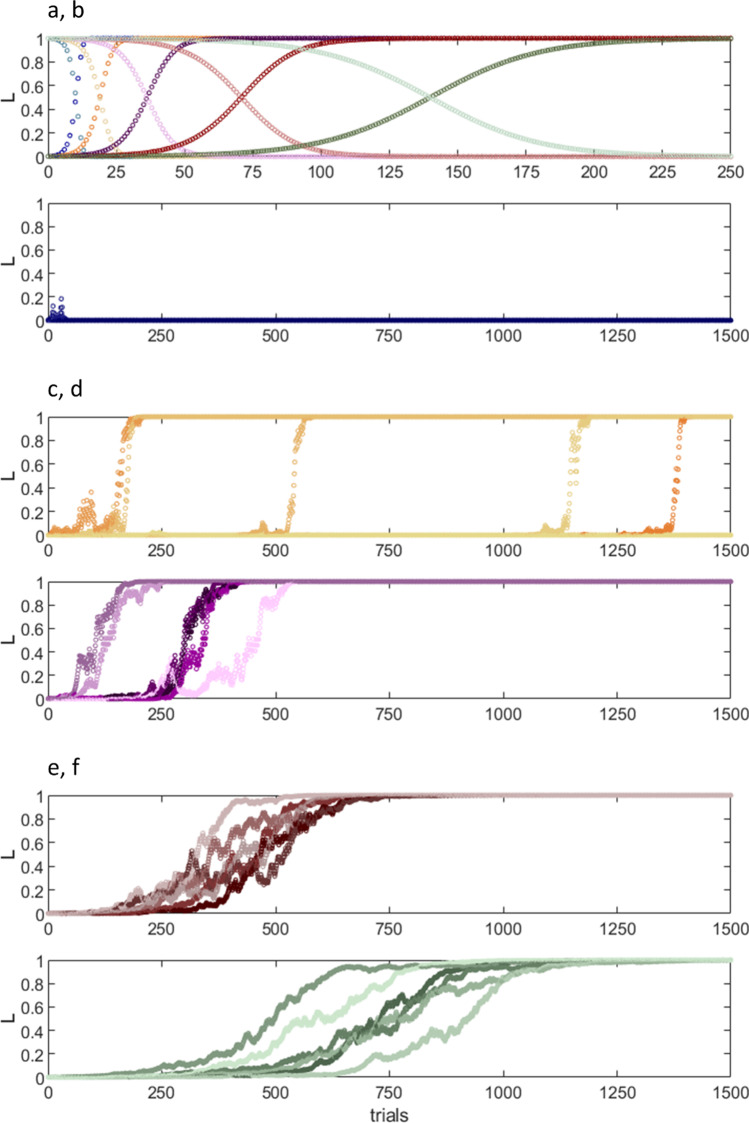
The effect of step size on learning. Parallel fibre synaptic activation teaches synaptic change. The direction of change depends on whether activation is paired with a climbing fibre signal. **a** Hypothesised learning and extinction curves for the parallel fibre synapse, with trials all paired and all unpaired respectively, varying the learning constant, $k$, in a sigmoidal function. $k$ = 0.8, 0.4, 0.2, 0.1, and 0.05 from left to right. **b**–**f** illustrate the effect of varying $k$ when activations are not all paired i.e. $p$ ≠ 1 or 0, likely outside experimental conditions. To isolate the effect of $k$, $p$ is fixed ($P$_(paired)_ = 0.6). The colour code is the same as in (**a**). Unpaired trials teach extinction, i.e., training is a randomly concatenated sequence of up and down steps, for 1,500 trials. Each panel (**b**–**f**) shows six simulations. **b** $k$ = 0.8. Despite the highest value of $k$, there is no learning. **c** $k$ = 0.4. Learning is explosive, but its onset is random. **d** $k$ = 0.2. The end result is reliable but the onset is still arbitrary and erratic. **e** $k$ = 0.1. There is reliable, consistently paced learning. **f** Learning is at longer latency with no improvement of consistency

The effect of changing the learning coefficient $k$ is shown in Fig. 2. This suggests a reason that learning needs repetition, so that in conditioning studies it may take several blocks of trials. A too large value yields unpredictable results when trials are paired with a probability *p* < 1, especially mid-range probabilities. This disappears with a smaller value of $k$. With small step size, learning takes longer but the outcome is consistent—either there is robust synaptic modification or there is no change. There is no other or intermediate outcome. A predictable effect that proceeds only in modest per-trial steps means that repetition can be used as the signal to learn. However, too-small steps would mean learning was very protracted (Fig. 2f). Presumably, at some point, protractedness would be inefficient and slow, so that per-trial step size is a trade-off between the advantages and disadvantages of using repetition as a signal. The value of $k$ that approximates the reported number of trials with all trials paired (Fig. 2a, red data) is the value used in the group learning simulation.

### Chaotic Real-World Training Variables

Learning in experimental all-trials-paired conditions—and, similarly, extinction training with all trials unpaired—is highly artificial. In real-world conditions, a number of variables contribute an unpredictable influence on synaptic modification, and these are outside cerebellar influence. Most synapses participate in more than one stored pattern. Each synapse participates in an independent random sample of patterns [because of decorrelation: 47]. Of that sample, the incidence of each pattern in any given rolling period of real-world training (i.e., set of trials), the ratio of the incidence of each to the others, and the sequence, are not random and do not occur with a fixed probability. Nor are they fixed or permanent; learning proceeds indefinitely and synapses are indefinitely plastic, new learning displacing old. To make the permutations more complex, synaptic change is reversible. Synaptic activation in the absence of a climbing fibre signal teaches modification in the opposite direction so that the recent ratios of paired to unpaired synaptic activations, and the sequence they occur in, are further variables. Finally, the amount of change induced by each training episode depends on the pre-episode weight of that synapse, and also which direction learning is in, in that trial. Taken together, the result is that each synapse receives an independently concatenated course of training which lacks a statistically reliable relationship with the learning outcome at any other synapse, indefinitely.

### Nucleo-olivary Feedback

Yet chaotic training variables must be converted into a predictable outcome (by default, because an arbitrary outcome would drive negative selection pressure). The—proposed—cerebellar solution involves inhibitory feedback to the inferior olive, the source of climbing fibre signals.

The output of a microzone, carried by Purkinje cells, is channelled down onto a smaller group of cells in deep nuclei, which forms part of the circuit and includes projection neurons which carry the output of the circuit. Each group sends an inhibitory (GABA) projection to the contralateral olivary complex [48, 49]. ‘A significant percentage of cerebellar nuclear projection cells, perhaps as many as half, provide GABAergic input to the inferior olive’ [50 p.14352]. Other estimates suggest 30–35% [51]. Most Purkinje cells contact GABAergic as well as glutamatergic nuclear projection neurons [52, 53]. About half of GABAergic and half of non-GABAergic terminals received by olivary cells are in glomeruli, where they co-terminate on gap junction-connected spines, and the rest on primary and intermediate dendrites [54].

Olivary groups innervate Purkinje cells with which they co-terminate in deep nuclei [55], so that they complete largely closed circuits [56–59]. Supplementary Materials contain a review of some of the evidence of closed circuits (Supplementary Materials 2, ‘A short review of the evidence of closed cerebellar circuits’).

Feedback is a learned response following training with a conditioning protocol, and inhibition is targeted and timed to block the signals that trained it [56]. Nucleo-olivary projection cells fire spontaneously. Elevation of firing of the feedback pathway coincides with a transient fall in Purkinje cell rates [60, 61]. The fall in the Purkinje cell rate is in part the result of coordinated inhibition of Purkinje cells by interneurons. There is accordingly, as training proceeds, a progressive swing in the balance between excitatory and inhibitory input to a Purkinje cell, as training weakens direct excitation and strengthens feed-forward inhibition of Purkinje cells via interneurons. GABAergic nuclear cells are reported to be sensitive to—indeed specialised to respond to—changes in the simple spike rate [62].

### Adding Nucleo-olivary Feedback to the Model

It is poorly understood what direct effect feedback has where it terminates and unknown what its function is. If climbing fibre instruction signals are binary (see above and [2]), it follows that feedback does not change what they teach, so that feedback is itself binary—not the firing rate of the feedback signal per se, but in its effect—such that, in any trial, feedback is either sufficient to block the climbing fibre signal in that trial or it is not.

In what would otherwise be a paired trial, but where the climbing fibre signal is blocked by feedback, the direction of learning is reversed. Using the Heaviside function, $H(x)$, we might represent the presence, or not, of a teaching signal as:

$${T}_{n}=H\left(f\langle r\rangle -\langle r{L}_{n-1}\rangle \right)$$

where $r$ is parallel fibre signal frequency, such that, at each synapse, $r{L}_{n-1}$ is the product of parallel fibre signal frequency and synaptic weight, and $\langle r{L}_{n-1}\rangle$ is the average of the products. A rising value of $\langle r{L}_{n-1}\rangle$ accompanies increasing depression of the Purkinje cell rate and corresponding disinhibition of the feedback pathway.^1^$f\langle r\rangle$ is the threshold where a rising value of $\langle r{L}_{n-1}\rangle$ blocks olivary discharge, so that there is no climbing fibre signal in that trial, determined on a trial-by-trial basis. So ${L}_{n}$ is the sum of ${L}_{n-1}$ and the product of

$$k\left(\left[ H\left(f\langle r\rangle -\langle r{L}_{n-1}\rangle \right) \right]-{L}_{n-1}\right) and\left\{\begin{array}{c}{L}_{n-1}, {T}_{n}=1\\ {1-L}_{n-1}, {T}_{n}=0\end{array}\right.$$

in trials where excitatory drive to the inferior olive is present.

What is the derivation of the threshold $f\langle r\rangle$ and of the relationship $f\langle r\rangle -\langle r{L}_{n-1}\rangle$?

Parallel fibres that contact a Purkinje cell make contact at an average of 1.24 synapses, that is, one and sometimes two [63, 64]. If we assume contact is at one synapse per cell, and that it is immaterial whereabouts on the Purkinje cell dendritic arbour contact is made,^2^ we can represent:

1. The effect of a signal on postsynaptic firing as a function of the product of signal frequency and synaptic weight, and overall, collectively, as the average of the products; and
2. Feedback signal frequency as a function of the Purkinje cell rate.

Point (2) requires the further assumption that the firing of microzone-grouped Purkinje cells is concerted, so that all nuclear cells receive the same effect although they each receive input from a different sub-group of Purkinje cells. This condition is met: see the discussion.

A climbing fibre signal is blocked where inhibition of an olivary group prevails against excitation that normally triggers a signal. Presumably, and in the model, the necessary strength of feedback is proportionate to excitation of the group, so that strong excitation must be met by proportionately stronger feedback, for example.

It is poorly understood what controls frequency modulation of excitatory input to the inferior olive and what function that might have. The model addresses this by predicting that mossy fibre activity that drives input to a microzone and input to the inferior olive group that forms part of the same circuit is at related rates. This allows the threshold to be automatically adjusted on a trial-by-trial basis, so that it is proportionate on each occasion to a function of rates received as input to the system. Thus, incidentally, the strength of drive to the inferior olive influences the learning outcome notwithstanding that it is not reflected in the firing signature of climbing fibre signals themselves.

The next two sections discuss, respectively, the physiological and computational mechanisms that provide the necessary relationship with mossy fibre rates.

### The Physiological Basis to Predict a Relationship

Stellate cells in the C3 region of the cerebellar cortex, which is involved in forelimb movement, respond (by firing) exclusively to stimulation of an associated discrete region of the body surface, or receptive field [8, 65–67], and not to stimulation of other fields. It is thought that this stimulation drives paired input which trains potentiation of the parallel fibre-stellate cell synapse, consistent with in vitro evidence [6, 68]. Input to stellate cells evoked by stimulation of other fields is to untrained synapses, where it is therefore without effect [65]. Transmission at an untrained synapse is undetectable even at several 100 Hz (Henrik Jörntel, private correspondence dated 31 March 2017).

Receptive field-specific input to C3 circuits effectively extends modular cerebellar circuit wiring to the body surface. Stimulation evokes mossy fibre and climbing fibre signals that travel by different pathways to converge on the same vertical slice of the cerebellar cortex [69, 70]. On the whole, there is ‘a close correspondence [of terminal fields] between inputs conveyed by climbing fibres to the molecular layer [where they terminate on Purkinje cells] and those conveyed by mossy fibres to the underlying granular layer [where they terminate on granule cells]’ [71 p.677]. Thus learning is induced with a single peripheral stimulus which evokes paired input—not just the same form of stimulus but the same event. This ‘is a feature that seems to be observed across species and other parts of the cerebellar cortex (for example, crus II in the rat)’ [72, p.305].

So, mossy fibre rates received as direct input to a microzone, and rates received as input to the inferior olive, are correlated if and to the extent they reflect the same or correlated parameters of stimulation. This does not (of course) establish a correlation, but it provides a basis in evidence to predict one.

### The Computational Relationship

In terms of a computational relationship, the author has proposed previously that mean firing rates of concurrently active mossy fibres have a relatively straightforward and feasibly linear relationship with mean parallel fibre signal frequencies downstream, over a minimum number of randomly sampled parallel fibres, a threshold satisfied by the homeostatically regulated number received as input to a Purkinje cell [1]. 

If that proposal is correct, $\langle r\rangle$ is related to excitatory input rates to the cell group in the inferior olive which forms part of the same circuit, with coupled timing, allowing the feedback threshold to be set at a function of $\langle r\rangle$, $f\langle r\rangle$. In the model, the function is a coefficient < 1. The threshold must be exceeded by the mean of the products of $r$ and ${L}_{n-1}$ to block a climbing fibre signal in a paired trial. The model assumes that $\langle r\rangle$ scales linearly with excitatory input to the inferior olive. Linearity is not necessary—the actual relationship may be different—but has the merit of being both credible and convenient. So, $\langle r\rangle$ is a measure of excitatory input to the inferior olive, and $f\langle r\rangle$ can be taken as a measure of the strength of inhibitory feedback sufficient to tip the scales, where $\langle r{L}_{n-1}\rangle$ is high enough for a block, calculated trial by trial.

$\langle r{L}_{n-1}\rangle$ jointly represents learning at the parallel fibre synapse on Purkinje cells and on interneurons.

### Results

**Fig. 3:**
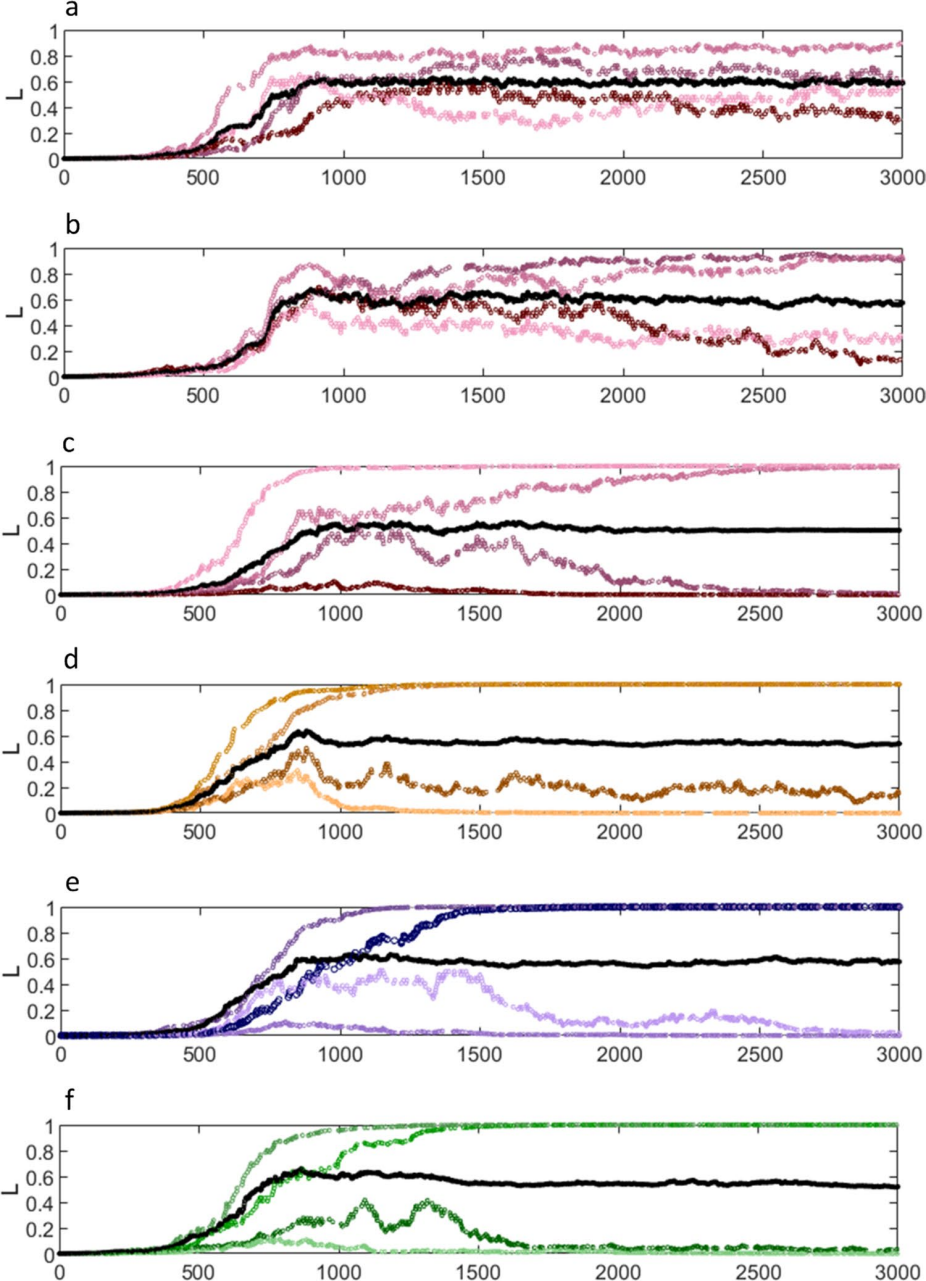
The effect of a collective feedback threshold on learning. A simulation of modification by training of 4 groups of four synapses, showing the proposed effect of nucleo-olivary feedback. Each synapse also participates in one of a further four groups, so that the groups can be visualised as a matrix. All 16 synapses are shown in (**c**)–(**f**), four in each panel. Grouped synapses receive climbing fibre signals together. Only one (randomly selected) group receives climbing fibre input per trial. There is a climbing fibre signal in an average of half of trials. In other trials, a synapse may either receive an unpaired input or else is silent, at random, but with a probability assigned to each synapse (which therefore determines the probability, at that synapse, that signals are paired). The ratio of paired to total trials is not intended to be physiological and is immaterial. The model simulates, rather, the change at a synapse trained with randomly concatenated paired and unpaired trials, where learning in paired trials is in groups that overlap. The black line is the average of the product of signal frequency and synaptic weight for the group. Following training, this is always stable at or near the feedback threshold, the value where climbing fibre signals are blocked in (what would otherwise be) paired trials. (**a**) and (**b**) are for comparison with (**c**)–(**f**). In (**a**), firing rates and the probability of paired trials are fixed (200 Hz; $p$ = 0.8). All groups are active, but data are shown for only one, as an example of the result. Learning gets ‘locked’ in a balance of mid-range but individually arbitrary weights (in a wide range). In (**b**), conditions are the same except that firing rates are randomly variable within synapse-prescribed limits. Synaptic modification appears to be in the early phase of slow polarisation, but the outcome remains inconclusive even after 3,000 trials. In (**c**)–(**f**) signals are each randomly assigned a firing rate in the range 100–300 Hz, and the probability that signals are paired is randomly generated for each synapse, such that each group contains a low ($p$ = 0.66–0.72), mid-low ($p$ = 0.72–0.78), mid-high ($p$ = 0.78–0.84), and high ($p$ = 0.84–0.9) probability. In all groups, following training, learning polarises and weights are stable. Fourteen out of sixteen synapses are pulled to the limits of the range. The other two are also stable but stranded at an intermediate weight to maintain the group average. At higher numbers, with larger groups, stranded weights are unnecessary

To bring all this together, it allows us to model the effect of training on a synaptic population whose members receive training that is randomly interlarded with extinction trials, and including an effect of nucleo-olivary feedback (Fig. 3). In each trial, synaptic activation is in one of a number of possible patterns generated in a random sequence. The ratio and sequence of paired and unpaired trials at each synapse is randomised with a synapse-assigned probability. Following training, in some nominally paired trials, the climbing fibre signal is blocked by feedback, reversing the direction of change. Firing rates of simulated parallel fibres that are active together vary from cell to cell, and the firing rate of each cell is variable on different occasions when it is active.

With this model, there is a well-predicted and stable outcome (Fig. 3c-f) of chaotic input to the system. The outcome is that stored patterns are always divided by training into a subgroup of synapses where modification saturates and a subgroup where the sign is reversed, in proportions that reflect the feedback threshold. To restate that, the synaptic outcome *within a pattern* polarises. A group threshold allows individual weights in the same pattern to be trained in different directions. A high feedback threshold means a proportionately high fraction saturate and low threshold a proportionately lower fraction. The proportion in the simulation approximates the reported proportion of strongly depressed parallel fibres on a Purkinje cell in the rat [23].

At low numbers used in the simulation, it is in some cases not possible to divide a group so that they all polarise, with the result that a synapse is stranded at an intermediate weight to maintain the group average (Fig. 3d). With larger numbers, outliers disappear because a split is always possible.

The probability of paired vs unpaired trials at all synapses is intentionally in the range that would teach change in the Fig. 2 simulation, to show that a split is always forced. To put this in another way, it is to show that, even with probabilities that would otherwise all teach synaptic change, *learning always generates the same split* in proportions which are dependent only on the feedback threshold. It does not matter to the outcome what exact probability of pairing synapses have individually. Any spread of probabilities will force the same split, given the same feedback threshold.

As noted early on, to get a stochastic result from randomly compounded training variables they must occur with a fixed probability, problematic because external variables are not random and do not have a fixed probability, and outside cerebellar control. The cerebellar solution is that individual probabilities, and individual synaptic outcomes, are immaterial. There is always the same split, regardless. In fact, non-fixed probabilities are used to destabilise the middle of the curve, so that weights migrate towards the limits of the range. A within-pattern variable probability of pairing at each synapse in fact *contrives* a predictable outcome (which is absent when variables are controlled—Fig. 3a), because the limits of the range are more stable than the middle. Uncertainty is a necessary ingredient of training because it increases instability at intermediate synaptic weights.

### Post Script: Purkinje Cell Dendritic Membrane Potential

Calcium entry into Purkinje cell dendrites is affected by local dendritic membrane potential. Membrane potential can be raised or lowered depending on whether the Purkinje cell receives or has recently received inhibition from interneurons [73, 74] or excitatory input from parallel fibres [75].^3^ An effect of membrane potential on learning is shown—in experimental conditions—by optogenetically suppressing calcium in VOR circuits [74].

Accordingly, following training, we might expect an arbour-wide—indeed microzone-wide—learned effect on calcium influx caused by feedforward inhibition of Purkinje cells. As the effect is to reduce calcium influx, it should preferentially affect low-affinity LTP in extinction trials. If so, this would mean that at higher values of ${L}_{n}$, extinction is in smaller per trial increments than acquisition (down steps are smaller than up).

To reflect this, the model includes a function which modifies step size, making down steps smaller as ${L}_{n}$ approaches 1

$${L}_{n}={L}_{n-1}+\left[\frac{1-{L}_{n-1}}{(1-b)(1-k)}\right]\left({T}_{n}-{L}_{n-1}\right)\left(1-{L}_{n-1}\right),\,{ T}_{n}=0;\,{L}_{n-1}>b$$

in extinction trials where ${L}_{n-1}$ exceeds $b$ (the new function in square brackets replaces $k$ and ${f}_{A}\left(x\right)$ disappears). This adjustment is made in the Fig. 3 simulation. This may help to explain the seeming ‘disappearance’ (really: reduction) of climbing fibre signals in trained circuits [48, 78]. Functionally, it causes $L$ to be ‘pulled’ more strongly towards a high value and increases the stability of memories once acquired.

## Discussion

Attempts to explain cerebellar function have been dominated by a motor learning role for climbing fibre signals since Marr’s original proposal [79]. The common features of traditional learning models are referred to as the supervised learning model [80]. The two main subclasses are the perceptron [originally: 12] and adaptive filter models [originally: 13].

The central premise of the supervised learning model is an assumption that learning is stored as long-term, finely graduated parallel fibre synaptic changes, trained under supervision of external instruction signals [12–15], provided by climbing fibres. This has the function that the naive Purkinje cell response to granule cell firing rates is displaced by a learned response controlled by incrementally acquired modification of synaptic weights. Weights are controlled by a learning algorithm. Learning is generally envisaged to correct behavioural errors, signalled by climbing fibre signals. A function of the organisation of Purkinje cells into microzones is absent, and firing of Purkinje cells is implicitly regarded as output of the system.

It is not always possible to directly compare models point by point, because they do not frame problems in the same way, or ask all the same questions, or make comparable proposals. To compare the present proposals to other models, it is therefore necessary to narrow down the field and the points under consideration. The writer has attempted to do that in two main ways. One is to restrict the focus to the scope of the paper: learning at the parallel fibre synapse. The other is to focus on a discussion of the evidence, after all the only real gauge of the strengths and weaknesses of a theory.

It is uncommon for computational papers to re-evaluate the evidence for core features of the model class they belong to, because it is considered unnecessary. The thinking is, implicitly, that early work established a platform of evidence that it would be needless to argue again. To compare claims to a basis in evidence, therefore, we must revisit the original justification and therefore the original proposals: for the perceptron, Albus [12, 81], and Fujita [13] for the adaptive filter model. Marr [79] is included because his proposals are in some ways fundamentally different.^4^ Table 1 contains a summary of a (non-exhaustive) comparison of main features of those models with each other and with the present proposals.

**Table 1:** Comparison on some main points of the principle learning models with each other and with the present model. Important note: this is not a comprehensive list but focussed on points most relevant to the present scope

	Marr	New model	Albus/perceptron	Fujita/adaptive filter
cf signal in teaching role	Yes	Yes	Yes	Yes
Sign of cf-trained pf-PC change	LTP	LTD	LTD	LTD
Binary or graded synaptic modification	‘Totally modified or totally unmodified’	Binary effect on collective transmission	Graduated	Graduated
Function of learning	Transmission of learned patterns	Transmission of learned patterns	Learned ability to group input patterns into predefined classes	Selectively weighted transmission of pf signals to give a ‘desired response’
Response depends on pattern	No	No	Yes	Yes
Functionally variable cf signature	No	No	Yes	Yes, contained in a time-varying discharge rate
Heterogeneous lessons	No	No	Yes	Analog signal
Unit of learning and memory	PC	Microzone	PC	PC
cf-trained learning at pf-MLI synapse	No	LTP	Yes, in different directions on outer and deeper level cells	Yes
Learning algorithm	No	No	Yes	Yes—assumed criterion of system performance is the mean square error
Physiological derivation of synaptic learning function	No function	Yes	No	No
Output of the model	Single PC firing rate*	Functionally indivisible (i) learning and (ii) behaviour of the circuit	Single PC firing rate**	Single PC firing rate
What codes PC firing?	pf rates: data packaged in single signals, as ‘codons’	pf rates: data indivisibly coded in all input	pf synaptic weights	pf synaptic weights
Plastic outcome coupled to training variables?	No proposed physiological or computational mechanism that sets weights	No	Yes	Yes
PC-nuclear anatomical contact ‘rules’	Outside cortex-only scope	Functional and integrated	Not included	Not included
Nucleo-olivary feedback	Outside cortex-only scope	Functional and integrated	Not included	Not included
Function of unknown patterns	None	Self-inhibition by the circuit of its own output	No unknown patterns	None: an effect of noise is eliminated by training
What limits pattern memory capacity?	Inhibition must block a response to random patterns	Conceivably no limit	Ultimately, overlap causes classification errors	Pattern memory is not the function of learning
Majority pf-PC synaptic ‘silence’	Supports predicted synaptic modification	Supports predicted synaptic modification	At best, not supporting evidence	At best, not supporting evidence
Recorded firing linearly codes behavioural parameters	Consistent with binary weights	Consistent with polarised weights	Problematic	Problematic

^*^Marr assumes ‘that the central nervous system has a means of converting a signal in a Purkinje cell axon into’ a motor command [77 p.455]

^**^Albus briefly moots that heterogeneously trained PCs may code a motor sequence, but this does not form part of the model. The short section on firing of nuclear cells really only states in the form of mathematical symbols what inputs nuclear cells receive

All of the models, including the present proposals, share the idea that climbing fibres have a teaching function, and that teaching modifies parallel fibre synaptic transmission strength. Where they fundamentally differ is whether output of the system is ultimately encoded in input rates to the system or generated internally by a graduated effect of synaptic weights on signal transmission. In the present model, it is the first, with learning confined to the role of memory-operated gating. In the supervised learning model, it is the second.

A gate is not a new idea [79]. Marr’s original proposal was that the function of synaptic modification was to select learned patterns for transmission, while blocking transmission of other patterns, rather than to modify the postsynaptic effect. For that purpose, he envisaged that a synapse was ‘either totally modified or totally unmodified’ [79 p. 456]. It was only afterwards that he became associated with the idea of learning-adapted weights, added by Albus [12]. Albus combined Marr’s idea of parallel fibre synaptic learning under climbing fibre supervision with an algorithm for binary classification of images developed earlier by Rosenblatt [83].

The idea of training-graduated synaptic weights was later adopted by Fujita in his proposal that the cerebellum may work in a way that is analogous to an adaptive filter [13]. Adaptive filters are used to adjust the amplitude (e.g. voltage) of selected parts of a signal received as input, such as preferred (or unwanted) frequencies.^5^ Fujita saw this as fixing limitations of a model based on pattern recognition (which could only generate episodic output), by replacing it with one which received analog input and generated analog output. He assumed also that climbing fibre signals are analog, coded in a time-varying rate of discharge. In fact, while some mossy fibres fire continuously during movement, both granule cells [84–86] and climbing fibres [36, 38, 82] fire in short high-frequency bursts. Climbing fibre bursts are at an invariant intraburst rate [37] and separated by an unusually long refractory period. Fujita is candid that his model ‘is based upon a number of assumptions and simplifications’ [13 p. 202].

The perceptron and adaptive filter models importantly differ with Marr (and the present model). Marr saw learning as a way of selectively permitting transmission of remembered patterns. In the perceptron and adaptive filter models, it is used instead to change the Purkinje cell response, replacing control by input rates with control by a learning algorithm.

Evidence of a learning algorithm is circumstantial—while synaptic modification may be what we would expect to observe if the Purkinje cell is a perceptron or adaptive filter, it might equally well have other explanations. It is similarly unsubstantiated that the cerebellum is the physiological implementation of whatever the algorithm does—neither the perceptron nor adaptive filter can cite a physiological derivation of the algorithm.

They both, however, importantly predict fine-grained graduation of trained synaptic weight adjustments—the mechanism of pattern recognition. The evidence is unfavourable. Naturally occurring depression at the parallel fibre-Purkinje cell synapse seems to be widespread and uniformly severe. The large majority of synapses are strongly depressed, so-called ‘silent’ synapses making up as much as 80–85% [23]. This is not to say that depressed synapses are all equally or fully stripped of AMPA receptors, although some of them are [87]. But transmission is uniform in being compressed into a narrow range where there is ‘no detectable somatic response’ to granule cell stimulation [23 p.9676]. This is consistent with a high estimate of ‘electrically silent’ synapses made by parallel fibres activated by cutaneous stimulation [9]. Attempts have been made to explain the data [14, 15], but another explanation is that the evidence does not support the model.

Cell firing data are also a problem. In computational network models that use simulated synaptic weight adjustments to convert input values into proposed output of the system—the method used by the supervised learning model—‘it is often extremely difficult to find any obvious relationships between the output of the individual units [single cells] and any of the features of the input stream or the final response of the system….In contrast, the firing rate of many cerebellar neurons is a linear function of task-related parameters….This linear coding of task-related parameters has been found at all levels of the cerebellar circuit’ [80 p.239]. The authors^6^ provide thirty references for linear coding. That is, recorded firing of cerebellar cells conflicts with the way they are predicted to fire if there are graduated synaptic weights.

The present model does not have those problems because synaptic weights are not graduated. To the extent that the balance between inhibition and excitation of Purkinje cells is controlled by learning, the balance has two states, thereby removing interference with faithful transmission of rate-coded data. In fact, part of the function is precisely to remove an effect of weights on transmission.

The evidence of linear transmission is not limited to a correlation of firing and behavioural parameters. In self-paced mouse locomotion, interneurons reflect ‘granule cell input with linear changes in firing rate’ [86 p.6], and ‘locomotion-dependent modulation of the balance between excitation and inhibition [of Purkinje cell dendrites] generates depolarising or hyperpolarising dendritic *V*_m_ [dendritic membrane voltage] changes that linearly transform into bidirectional modulation of PC SSp [Purkinje cell simple spike] output’ [86 p.9].

Linear transmission supports the idea that weights do not modify (by graduating) transmission and therefore indirectly a gating role of memory. On the face of it, however, gating (like other models) still has a long recognised problem [12, 79]. This is that Purkinje cells receive inhibition from laterally positioned interneurons, so that inhibition of a Purkinje cell is driven by a largely different population of parallel fibres than the ones that make direct contact on it. The stellate cell main axon extends sagittally, so that in addition to contact on immediately flanking Purkinje cells, stellate cells also inhibit sagittally neighbouring Purkinje cells. Not all do so—there is a depth-dependent morphology gradient [25, 82, 88, 89]—but deeper-lying stellate cell axons can have a range of up to 450 µm [25], and basket cells may converge on a Purkinje cell from a still greater range that covers half a microzone [82, 90, 91]. This causes the issue that it is difficult to conceive (and intractable to model) how direct excitatory input to a Purkinje cell and feed-forward inhibition combine for a precise effect.

For Marr, the role of interneurons is to hold Purkinje cells under inhibitory, default restraint unless parallel fibres are active in a learned pattern. In order for direct excitation of a Purkinje cell to prevail over inhibition, it was necessary that a very high fraction (‘close to one’) of direct input was received at modified synapses, which he thought were strengthened by training. He predicted that synapses on interneurons were not plastic, for which he saw no need in this role.^7^ Albus proposed that training strengthens synapses on stellate cells, motivated by the performance enhancement that would be provided by a bidirectional learning outcome. To deal with lateral inhibition, he argues that strengthening should be confined to stellate cells in the outer molecular layer and that the direction of learning is reversed at deeper level, weakening synapses on deeper-lying stellate cells and on basket cells.^8^ Fujita predicts a balance between excitation and inhibition of Purkinje cells that is adjustable in both directions, but does not commit himself to any particular mechanism. To simplify feed-forward inhibitory pathways, Fujita assumes ‘one inhibitory interneuron for one Purkinje cell’ [13 p. 198].

Generally, there has been a limited appetite to re-engage with the anatomical evidence. Simplifying assumptions are preferred: for example, incorporating interneurons into a continuous, learning-modulated scale of net excitation/inhibition of Purkinje cells. In fact, the problem is not anatomy but a modelling assumption that control of single Purkinje cells is somehow individually coded in the signals they receive and that the goal is to explain how. The present proposals do not have that issue because Purkinje cells are not the unit of learning or coding.

Marr and Albus published before it was reported that Purkinje cells are organised functionally into microzones [92–95]. Fujita raises the possibility of scaling up the ‘one Purkinje cell model’ to a microzone model but says that it ‘does not explain any more functioning of the cerebellum than a single cell model’ [13 p. 203]. The focus was never reframed from ‘what controls Purkinje cells?’ to ‘why are they in groups?’.

The absence of a theoretical explanation of organisation of Purkinje cells into microzones meant there was no competition to displace the assumption that single Purkinje cells are the unit of learning and that ‘individual Purkinje cells most probably require specific error signals and learn heterogeneously’ [96 p.3]. This had the theoretical justification that heterogeneous instruction made the ‘computations’ more powerful that it was believed the cerebellum carried out, assuming the cerebellum worked as predicted by the traditional model.

The author has argued previously that the repeating microcircuit is the smallest functional unit in the cerebellum [2] and that information contained in firing rates is not in single signals but encoded in statistical properties of population activity [1]. For rate coding purposes, it does not matter which subgroup of cells is active or which of them fires at any particular rate. This allows information to be coded in any random sample of input to a microzone subject to a minimum sample size (unpublished data). With this model, there is an important coding function of the termination pattern of mossy fibres, which end in multiple, sagittally aligned terminal clusters^9^ [98–101], traditionally problem evidence for the single Purkinje cell model.

Climbing fibres do not terminate on a single Purkinje cell but on a variable number in the range 1–10, which therefore receive the same climbing fibre signals at the same time (at all climbing fibre synapses, as each Purkinje cell receives contact from only one climbing fibre). Moreover, climbing fibres that terminate on a microzone all discharge at the same time, so that microzone-grouped Purkinje cells receive instruction signals in volley, as one. Indeed, microzones are defined by their climbing fibre input. This would not in itself conclusively rule out bespoke lessons contained in the same climbing fibre volley. However, the data available about the signature of climbing fibre discharge does not convincingly support the idea of functionally variable instruction. On the contrary, the evidence makes a robust argument for the opposite view, that lessons do not vary. As that case has been made previously, it is not repeated here but appears (in a fuller-than-previously-published form) in Supplementary Materials 1. If correct, teaching signals are not bespoke, because they are not functionally variable.

In the present model, standardisation of instruction signals is not a lack of flexibility but functional, contributing to microzone-wide learning and a concerted effect of memory on microzone-wide behaviour. The climbing fibre projection, originating in the contralateral inferior olive and terminating with the footprint of a microzone, forms part of the ‘cerebellar’ circuit (see Supplementary Materials 2). The circuit is made up on its other two sides by the output of the microzone to the cell group in deep nuclei which includes the output cells of the circuit, and the inhibitory projection from that group back to the olivary group (reciprocated by climbing fibre collaterals).

Functionally indivisible circuit behaviour predicts and would explain seemingly anatomically indivisible output of microzone-grouped Purkinje cells to a nuclear group. Purkinje cells outnumber nuclear cells around 10:1, a Purkinje cell makes individually strong contact on 4–5 nuclear cells, and each nuclear cell receives contact from 30 to 50 Purkinje cells [rats: 102]. There is no known internal organisation of the output of a microzone to a nuclear group [Bengtsson and Jorntell in 103 p. 663]. The traditional model does not explain how heterogeneously trained output of Purkinje cells is mixed down onto nuclear cells and what function that has. The present proposals do not have that problem because learning is not heterogeneous. Unlike the single-cell model, microzone-wide memory is reconciled with what appears to be random sampling by nuclear cells of Purkinje cells [2].

Nucleo-olivary feedback is also poorly represented in traditional learning models. In a recent and authoritative review of the core features and computational principles of supervised learning models, inhibitory feedback to the inferior olive is not mentioned [80]. Feedback has an important and functional part in the present proposals.

Finally, it is not clear that a learning algorithm could in fact learn from real-world variables which are outside cerebellar control, not random and do not occur with fixed probabilities, and which are compounded in training in a manner which also has those features. This is problematic because overlap of paired patterns, and paired with unpaired patterns, means each synapse is likely to receive a unique course of training which does not have a reliably predictable relationship with any other synapse, or with any particular pattern in which it participates.

In the present proposal, that is not an issue because training variables do not determine the synaptic outcome. Instead, the outcome is fixed—always the same—and because it is fixed, it is predictable. As a result, all ‘remembered’ patterns receive the same effect on transmission at the functional scale that codes information.

There is no learning-controlled graduated balance between excitation and inhibition of Purkinje cells. Instead, it has two functional states—a switch—at synaptic, whole cell and microzone level. It is unnecessary that depressed synapses are equally or all fully blocked, although some of them are [87], because transmission is by an effectively random sample of weights and therefore normalised over a scale threshold. Indeed, in the new model, variably graded synaptic transmission strength—the mainstay of the supervised learning model—would cause dysfunctional interference with faithful transmission of rate codes.

The author has previously proposed that intelligent anatomy of functionally unitary circuits implements a memory-controlled gate [2]. The present proposal is a complementary and integrated but separate mechanism. The two together orchestrate a unitary response of the functionally undivided circuit.

Finally, it is a feature of the learning function that it models induction without modelling the underlying molecular pathways. The stripped-down character of the function might be likened to the neck of an hourglass. The top represents events outside the cerebellum which generate uncontrolled training variables. The bottom represents subcellular events involved in making parallel fibre synaptic plastic changes. The narrowness of the neck represents sparsening of variables that affect calcium amplitude. The narrowness of the neck, and role of calcium in induction, mean that control of calcium predicts the synaptic learning outcome, creating selection pressure that eliminates other variables.

## Supplementary Information

Below is the link to the electronic supplementary material.

Supplementary file1 (DOCX 69.3 KB)

## Footnotes

1 This is because ${L}_{n-1}$ represents weakening of parallel fibre-Purkinje cell synaptic transmission and strengthening of parallel fibre-interneuron transmission.

2 Reasonable, because it is physiologically justified by the distribution of spines and the randomised distribution of active cells among the parallel fibre population that make contact.

3 Possibly, membrane potential also depends on whether the cell is in an up or a down state—however, in the presence of Purkinje cell membrane bistability in awake, behaving animals is contentious, that is, probably absent (76.Schonewille, M., et al., *Purkinje cells in awake behaving animals operate at the upstate membrane potential.* Nat Neurosci, 2006. **9**(4): p. 459–61; author reply 461, 77.Yartsev, M.M., et al., *Pausing purkinje cells in the cerebellum of the awake cat.* Front Syst Neurosci, 2009. **3**: p. 2.).

4 Marr’s evidence, incidentally, was mainly a recently published book by Eccles and others [80.Eccles, J.C., M. Ito, and J. Szentágothai, *The cerebellum as a neuronal machine*. 1967, Berlin, New York etc.: Springer-Verlag. 335 p.].

5 Adaptive filters are used, for example, to remove mains hum from a heart trace and feedback from an amplified acoustic signal.

6 In fact advocates of the supervised learning model, but also of a balanced review.

7 Marr allows that operation in this way might lead you to expect (counter to the evidence) that each Purkinje cell would be partnered with a single molecular layer interneuron of the same size and shape, so it would sample the same parallel fibre activity.

8 Albus does not explain (or model) how parallel fibre-interneuron synaptic adjustment is confined only to the ‘immediate vicinity of the Purkinje cell’ under instruction or how instruction teaches weight change in different directions at superficial and deeper level.

9 A mossy fibre axon may give rise to several terminal clusters, aligned sagittally, the direction of the long axis of a microzone, separated by a minimum but otherwise apparently arbitrary distance, so that branch endings are randomly spaced in a sagittal band.
