# Supplementary material for: Gating by Memory: a Theory of Learning in the Cerebellum
Source: Cerebellum. 2021 Nov 10;21(6):926–43. doi: 10.1007/s12311-021-01325-9 (PMC9596590; doi:10.1007/s12311-021-01325-9)
Supplement: Supplementary file 1 — Supplementary file1 (DOCX 69.3 KB) [file 12311_2021_1325_MOESM1_ESM.docx]

SUPPLEMENTARY MATERIALS

**SM 1 Climbing fibre instruction signals are not functionally variable**

Climbing fibres discharge is in short bursts of spikes, originally reported to have an all-or-nothing signature [1]. The number of spikes is variable but in a small range and unpredictable on any particular occasion [2]. There has been a more recent move to argue – and is now probably the mainstream view [3-5] – that climbing fibre signals teach a graded lesson that depends on the number of spikes in a burst. These materials argue for a rehabilitation of the all-or-nothing view, and that the evidence is robust. A shorter form has appeared previously [6].

Discharge is at invariant, high frequency [7, 8]. Over many bursts, the number of spikes correlates statistically with the timing of discharge relative to the phase of inferior olive subthreshold oscillation [8], leading to the idea that the number of spikes may code oscillations, teaching graded parallel fibre synaptic change. The number of spikes in a burst was later reported to correlate with the amplitude, but not the phase, of oscillations [9]. The discrepancy is unresolved.

Oscillations do not predict the number of spikes in any given case. Caution has been urged, even by supporters of a graded signal. The ‘number of spikes per CF [climbing fibre] burst was quite variable from one burst to the next and … the changes in burst size for any given situation were small (<1 spike per burst) and could only be detected in the average as a slight probability bias toward generating more bursts with many (>4) or few (1) spikes’ [10 p.4]. Nor has a correlation been shown to be functional. If oscillations code anything it is unknown what it is.

The number of spikes is very modest. The average number of spikes in a burst is 2-3 and the range is 1-6 [8, 9]. The number is further reduced because at least 3 spikes are needed to induce LTD [11], and because the number is inversely correlated to the amplitude of oscillations [9], which is larger among functionally-grouped, strongly gap-junction-connected cells.

Moreover, spikes do not propagate reliably. Spikes are generated in the initial axonal segment. Some axonal spikes fail to propagate far (>125 μm), so that what is initially a group of 4 (say) can become a group of 3, or two. The first spike always propagates, and the others propagate with variable probability (range *p* = 0.66-0.89) [8]. The probability depends on their position in a burst. Transmission failure is at random. The probability of redacted spikes depends on the original size of the group – for example, an average of 2 out of 3 five-spike bursts are redacted. The unpredictable failure of spikes to propagate means that if information is coded in the number of spikes initially generated, it is unreliably transmitted.

Burst size does not code signals received as input to the inferior olive – ‘The mean number of spikes … [was] independent of the stimulus intensity’ [2 p.201]. This was found whether depolarisation is just over threshold or stronger, and has never been refuted. So, input data coded as excitatory rates are not represented in the number of spikes, even before spikes are lost to transmission failure. Nor is a graded lesson coded in the intraburst rate. Interspike intervals are highly reliable, such ‘that the timing of spikes within a burst in the olivary axon is highly stereotyped…, with only the number of spikes varying’ [8 p.392].

So: graded climbing fibre training signals would need to be coded in a very modest number of spikes. Moreover, the number of spikes does not code signals that drive firing. Even if it did, spikes are not reliably transmitted – targets would receive the wrong lesson more often than not. The interspike interval is always the same, so information is not coded in the spike rate within a burst. Contrary to the graded view, all of this suggests more naturally that the climbing fibre signature is narrowly *constrained* in form, and that the all-or-nothing view is the correct interpretation of the evidence.

**SM 2 A short review of the evidence of closed cerebellar circuits**

Most Purkinje cells that innervate a nuclear group originate in the same microzone or the same functional but dispersed group of microzones which form part of a multizonal circuit [12, 13]. Olivary cells contact Purkinje cells with which they co-terminate collaterally in deep nuclei [14] [see also 15, 16]. Collaterals are reciprocated by the inhibitory nucleo-olivary projection [17, 18], forming what are thought to be substantially closed circuits [16, 19-21]. Segregation is not perfect but organisation into circuits is well established. ‘CF [climbing fibre], olivonuclear, and corticonuclear axons project in a map-like fashion: neighbouring neurons in one region (olive, cortex, or deep nucleus) innervate neighbours in the other two regions’ [22 p.14]. In part of the C1 zone, for example, whose output is to the anterior interpositus nucleus and whose circuits control hind limb movements in rats, ‘a fine grain topography exists’ [23 p.16440].

Possibly, this does not hold for all circuits. Purkinje cell termination patterns are less focussed in the fastigial nucleus, for example [24]. Also, output of a microzone may be received by more than one nuclear location [13], probably as the result of Purkinje cell collateralisation [25]. Nonetheless, closed circuit organisation seems to be broadly preserved [13].

Deep nuclei integrate the descending input of Purkinje cells with collateral inputs from mossy fibres and climbing fibres to generate the output of the cerebellum [26, 27]. Contact by Purkinje cells on nuclear cells is preferentially somatic [28, p.3443] and individually strong [29], which may allow them to gate an effect of excitatory inputs, which are mainly (75%) dendritic [30]. However, not all mossy fibres send collateral projections to deep nuclei. Only 1 out of 15 mossy fibres originating in dorsal column nuclei, for example, had a collateral which terminated in a deep nucleus [31], and there is generally light collateral input to the dentate nucleus, such that the inhibitory input from Purkinje cells plays the primary role in controlling the dentate output [32].

Nucleo-cortical feedback is discussed in the main text. In addition to activation of the feedback pathway by disinhibition in the conditioned response, feedback cells may also receive collaterals of excitatory nuclear projection neurons which carry the main output of the circuit and which are disinhibited at the same time [33].

Evidence of group coded firing of functionally-grouped Purkinje cells is difficult to obtain but now emerging. In the flocculus, the averaged firing rate of functionally-grouped Purkinje cells (approximated by binning spikes across the population of recorded cells) has a linear, rapidly-translated relationship with eye movement [34], with 3-5 ms temporal precision. Likewise, activity of groups of Purkinje cells in the oculomotor vermis precisely matches the metrics of saccades [35, 36]. The significance (in this paper) is that control is rate coded in group activity, arguing for functionally unitary circuits. Rate control is supported by modelling [37].

REFERENCES

1. Eccles, J.C., R. Llinas, and K. Sasaki, *The excitatory synaptic action of climbing fibres on the Purkinje cells of the cerebellum.* J Physiol, 1966. **182**(2): p. 268-96.

2. Crill, W.E., *Unitary multiple-spiked responses in cat inferior olive nucleus.* J Neurophysiol, 1970. **33**(2): p. 199-209.

3. Hansel, C., *Reading the clock: how Purkinje cells decode the phase of olivary oscillations.* Neuron, 2009. **62**(3): p. 308-9.

4. Rasmussen, A., *Graded error signals in eyeblink conditioning.* Neurobiol Learn Mem, 2019. **170**: p. 107023.

5. Zang, Y. and E. De Schutter, *Climbing Fibers Provide Graded Error Signals in Cerebellar Learning.* Frontiers in Systems Neuroscience, 2019. **13**(46).

6. Gilbert, M. and R.C. Miall, *Gating by Functionally Indivisible Cerebellar Circuits: a Hypothesis.* The Cerebellum, 2021bb.

7. Maruta, J., R.A. Hensbroek, and J.I. Simpson, *Intraburst and interburst signaling by climbing fibers.* J Neurosci, 2007. **27**(42): p. 11263-70.

8. Mathy, A., et al., *Encoding of oscillations by axonal bursts in inferior olive neurons.* Neuron, 2009. **62**(3): p. 388-99.

9. Bazzigaluppi, P., et al., *Olivary subthreshold oscillations and burst activity revisited.* Front Neural Circuits, 2012. **6**: p. 91.

10. Najafi, F. and J.F. Medina, *Beyond "all-or-nothing" climbing fibers: graded representation of teaching signals in Purkinje cells.* Front Neural Circuits, 2013. **7**: p. 115.

11. Rasmussen, A., et al., *Number of spikes in climbing fibers determines the direction of cerebellar learning.* J Neurosci, 2013. **33**(33): p. 13436-40.

12. Apps, R. and M. Garwicz, *Anatomical and physiological foundations of cerebellar information processing.* Nat Rev Neurosci, 2005. **6**(4): p. 297-311.

13. Pantò, M.R., et al., *Corticonuclear projections of the cerebellum preserve both anteroposterior and mediolateral pairing patterns.* European Journal of Neuroscience, 2001. **13**(4): p. 694-708.

14. Llinás, R.R., K.D. Walton, and E.J. Lang, *7. Cerebellum*, in *The synaptic organization of the brain*, G.M. Shepherd, Editor. 2004, Oxford University Press: Oxford.

15. Ruigrok, T.J., *Cerebellar nuclei: the olivary connection.* Prog Brain Res, 1997. **114**: p. 167-92.

16. Bengtsson, F. and G. Hesslow, *Cerebellar control of the inferior olive.* Cerebellum, 2006. **5**(1): p. 7-14.

17. Hesslow, G. and M. Ivarsson, *Inhibition of the inferior olive during conditioned responses in the decerebrate ferret.* Exp Brain Res, 1996. **110**(1): p. 36-46.

18. Bengtsson, F., P. Svensson, and G. Hesslow, *Feedback control of Purkinje cell activity by the cerebello-olivary pathway.* Eur J Neurosci, 2004. **20**(11): p. 2999-3005.

19. Shinoda, Y., et al., *The entire trajectory of single climbing and mossy fibers in the cerebellar nuclei and cortex.* Prog Brain Res, 2000. **124**: p. 173-86.

20. Pijpers, A., J. Voogd, and T.J. Ruigrok, *Topography of olivo-cortico-nuclear modules in the intermediate cerebellum of the rat.* J Comp Neurol, 2005. **492**(2): p. 193-213.

21. Fujita, H. and I. Sugihara, *Branching patterns of olivocerebellar axons in relation to the compartmental organization of the cerebellum.* Front Neural Circuits, 2013. **7**: p. 3.

22. Ozden, I., et al., *Widespread state-dependent shifts in cerebellar activity in locomoting mice.* PLoS One, 2012. **7**(8): p. e42650.

23. Cerminara, N.L., et al., *Structural basis of cerebellar microcircuits in the rat.* J Neurosci, 2013. **33**(42): p. 16427-42.

24. Sugihara, I., et al., *Projection of reconstructed single Purkinje cell axons in relation to the cortical and nuclear aldolase C compartments of the rat cerebellum.* J Comp Neurol, 2009. **512**(2): p. 282-304.

25. De Zeeuw, C.I., et al., *Projections of individual Purkinje cells of identified zones in the flocculus to the vestibular and cerebellar nuclei in the rabbit.* J Comp Neurol, 1994. **349**(3): p. 428-47.

26. Bengtsson, F. and H. Jörntell, *Specific relationship between excitatory inputs and climbing fiber receptive fields in deep cerebellar nuclear neurons.* PLoS One, 2014. **9**(1): p. e84616.

27. Bengtsson, F., C.F. Ekerot, and H. Jorntell, *In vivo analysis of inhibitory synaptic inputs and rebounds in deep cerebellar nuclear neurons.* PLoS One, 2011. **6**(4): p. e18822.

28. Uusisaari, M. and E. De Schutter, *The mysterious microcircuitry of the cerebellar nuclei.* J Physiol, 2011. **589**(Pt 14): p. 3441-57.

29. Person, A.L. and I.M. Raman, *Purkinje neuron synchrony elicits time-locked spiking in the cerebellar nuclei.* Nature, 2012b. **481**(7382): p. 502-505.

30. de Zeeuw, C.I. and A.S. Berrebi, *Individual Purkinje cell axons terminate on both inhibitory and excitatory neurons in the cerebellar and vestibular nuclei.* Ann N Y Acad Sci, 1996. **781**: p. 607-10.

31. Quy, P.N., et al., *Projection patterns of single mossy fiber axons originating from the dorsal column nuclei mapped on the aldolase C compartments in the rat cerebellar cortex.* J Comp Neurol, 2011. **519**(5): p. 874-99.

32. Ishikawa, T., et al., *Releasing dentate nucleus cells from Purkinje cell inhibition generates output from the cerebrocerebellum.* PLoS One, 2014. **9**(10): p. e108774.

33. Svensson, P., F. Bengtsson, and G. Hesslow, *Cerebellar inhibition of inferior olivary transmission in the decerebrate ferret.* Exp Brain Res, 2006. **168**(1-2): p. 241-53.

34. Payne, H.L., et al., *Cerebellar Purkinje cells control eye movements with a rapid rate code that is invariant to spike irregularity.* Elife, 2019. **8**.

35. Herzfeld, D.J., et al., *Encoding of action by the Purkinje cells of the cerebellum.* Nature, 2015. **526**(7573): p. 439-42.

36. Herzfeld, D.J., et al., *Encoding of error and learning to correct that error by the Purkinje cells of the cerebellum.* Nat Neurosci, 2018. **21**(5): p. 736-743.

37. Abbasi, S., et al., *Robust transmission of rate coding in the inhibitory Purkinje cell to cerebellar nuclei pathway in awake mice.* PLoS computational biology, 2017. **13**(6): p. e1005578-e1005578.
